# Habituation is not neutral or equal: Individual differences in tolerance suggest an overlooked personality trait

**DOI:** 10.1126/sciadv.aaz0870

**Published:** 2020-07-08

**Authors:** Andrew T. L. Allan, Annie L. Bailey, Russell A. Hill

**Affiliations:** 1 Department of Anthropology, Durham University, Dawson Building, South Road, Durham DH1 3LE, UK.; 2 Primate and Predator Project, Lajuma Research Centre, PO Box 522, Louis Trichardt 0920, South Africa.; 3 Department of Zoology, University of Venda, Private Bag X5050, Thohoyandou 0950, South Africa.

## Abstract

In behavioral studies, observer effects can be substantial, even for habituated animals, but few studies account for potential observer-related phenomenon empirically. We used wild, habituated chacma baboons to explore two key assumptions of behavioral ecology (i) that observers become a “neutral” stimulus and (ii) that habituation is “equal” across group members. Using flight initiation distance (FID) methods within a personality paradigm, the behavioral responses of baboons suggested that observers were not perceived as neutral but instead viewed as a high-ranking social threat. Habituation was also not equal across group members, with repeatable individual differences more important than contextual factors (e.g., habitat) in determining the distance at which baboons visually oriented or displaced from observers. A strong correlation between individual visual tolerance and displacement tolerance (i.e., convergent validity) indicated a personality trait. We offer several suggestions for how to account for these factors and minimize potential bias in future studies.

## Teaser

Wild habituated baboons show individual differences in tolerance to researcher proximity that may undermine behavioral data.

## INTRODUCTION

Habituation has been referred to as “a process that leads to decreased responsiveness to a stimulus” [see page 255 of (*1*)]. In behavioral ecology, habituation has been used to reduce the risk perception that wild animals have toward human observers, with the outcome of such processes or “full habituation” described as “individual accepts humans (and apparently ignores them) at close range during all activities; they appear calm when they are alone with humans and are relatively easy to follow while travelling” [see page 164 of (*2*)]. This allows researchers to conduct behavioral observations under the assumption that study subjects have lost their fear of human observers and view them as a neutral stimulus (*3*, *4*). The wealth of literature using data collected from behavioral observations on habituated animals suggests that this process is tried and tested in numerous species. However, recent work strongly suggests that observer presence is unlikely to have a neutral effect on study animals. Welch and colleagues (*5*) found that bat-eared foxes (*Otocyon megalotis*) increased vigilance during the early stages of focal observations, while reef fishes had significantly higher rates of cleaning interactions when data were collected using video observations (divers absent) versus direct observations by divers (*6*). Nevertheless, while the concept of observer neutrality has received discussion across a range of species typically exposed to direct observations [e.g., baboons (*7*), macaques (*8*), bonobos (*9*), and meerkats (*10*)], overall, there is a lack of empirical research focusing on observer neutrality in habituated systems.

The outcome of habituation processes has been referred to as tolerance, with highly tolerant animals consistently allowing closer approaches by humans without adjusting their behavior or fleeing and vice versa for highly intolerant animals (*1*). This suggests that the tolerance outcome of habituation processes exists along a spectrum, allowing tolerance to vary across individuals, groups, and species. Hanson and Riley (*8*) highlighted an observable difference in tolerance across two study groups of moor macaques (*Macaca maura*), further suggesting that habituation is a flexible, context-dependent spectrum of heightened observer tolerance. Beyond group or species differences in tolerance, the wider assumption that the outcome of habituation can be considered “equal” across individuals within groups (and across solitary individuals) remains untested empirically. If subtle variation in tolerance levels is overlooked, then a key driver of behavioral patterns is also missed, which could have far-reaching implications for behavioral research.

If there are consistent interindividual differences in tolerance to human observers and within-individual tolerance is consistent through time and in response to multiple contexts and situations, then tolerance would satisfy the conditions for being classed as a personality trait (*11*). If tolerance is a personality trait, then it suggests that three implicit assumptions concerning habituated animals may not be entirely valid, namely, (i) that observers are considered neutral, (ii) that habituation (i.e., tolerance) is equal across study animals, and (iii) that observers play little to no role in the behaviors that they record. Here, we explore these assumptions within a tolerance-personality paradigm using a group of Afromontane chacma baboons (*Papio ursinus griseipes*) as a model species.

As individual tolerance has yet to receive attention from a personality perspective, identifying an ecologically valid measure is of critical importance. The factors influencing the habituation process have received some attention in primatology (*12*). Behaviors such as observer-directed aggression (*13*) and self-directed behaviors (*14*) were both found to decrease over the course of habituation in white-headed capuchins (*Cebus capucinus*) and vervet monkeys (*Chlorocebus pygerythrus*), respectively. Baboons (*Papio* spp.) and rhesus macaques (*Macaca mulatta*) were shown to exhibit altered behavioral patterns between observer presence and absence treatments, but effects differed across species and sex (*15*). In contrast, there was no evidence found that observer presence influenced the activity patterns, ranging behavior, or proximity to neighboring conspecific groups of wild habituated white-faced capuchins (*C. capucinus*) (*16*). While understanding the habituation process and behavior in the presence/absence of observers is important, the approach does not offer a methodological framework to assess any individual personality factors relating to tolerance to observers or the real-time implications of human presence on the behavior of study animals.

Personality types in wild habituated baboons (*Papio ursinus ruacana*), specifically boldness and anxiousness, have been demonstrated in response to novel food items and model predators, respectively (*17*). Both traits were investigated using both categorical/binary responses (e.g., back away or tail flag) and continuous measures (e.g., handling time) under experimental conditions (*17*), with each process relying on individuals encountering and responding to static stimuli. However, tolerance centers around an individual’s tendency to endure the behavior of a human observer without altering behavioral patterns, and as a result, static stimuli are inappropriate. Instead, measures need to mimic the stimuli of human observers moving around the environment concurrent to study animals (*18*).

Quantitative and objective measures are thus required to infer tolerance (*8*). Flight initiation and alert distances offer a methodological process that produces continuous measures (i.e., distance) in response to an observer walking toward study animals (*19*). Optimal escape theory predicts that the point at which prey decides to flee from an approaching predator, otherwise known as flight initiation distance (FID), is governed by a trade-off between the risk of being predated upon and the benefits of staying to engage in any fitness-enhancing activity; increasing perceived risk of predation should thus lead to increased FID (*20*, *21*). FID methodology has previously been used as a proxy for measuring the personality trait boldness (*22*, *23*), in each case assessing individual repeatability in FID responses as a proxy for the boldness trait. Inferring boldness using FID methodology is dependent on the assumption that human approachers are considered threatening or novel, but FID approaches on habituated animals are unlikely to be an ecologically valid measure of an animal’s propensity to engage in risky, innovative, or novel behaviors (i.e., boldness). Instead, it is likely to measure the propensity of individuals to endure human actions without altering behavior (i.e., tolerance). A clear tolerance measure that can be derived from FIDs is “displacement tolerance,” the propensity of an animal to endure proximity to a human observer without moving away. Tied to this is the additional measure of “detection distance” that should also provide an ecologically valid method for inferring visual tolerance, i.e., individual tendency to visually orient or to resist directing looking toward an approaching observer.

**Fig. 1:**
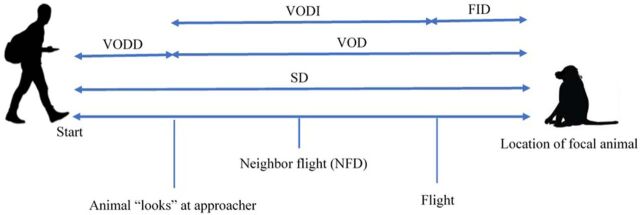
FID procedure and measurements. This highlights the distance variables that can be measured as a function of the focal animal’s behavioral responses. Start distance (SD), visual orientation distance (VOD), VOD delay (VODD), VOD interval (VODI), flight initiation distance (FID). Adapted from (*57*).

In this study, we used FID methodology to explore whether tolerance can be considered a personality trait in Afromontane chacma baboons exploring individual repeatability for two specific behaviors: FID and visual orientation distance (VOD). FID refers to the distance at which individual baboons were displaced by approaching humans (Fig. 1), while VOD refers to the distance at which individual baboons oriented their looking behavior toward the observer as a result of their approach. Typically, FID studies will record a vigilance or alert distance (*19*). We use VOD as an equivalent for alert distance; the difference in terminology is based on the constraint that this study focuses on habituated primates that are aware of our presence before initiating approaches, and so, visual orientation, as opposed to alert, best describes the behavior of the focal animal looking toward the approaching observer.

To be considered a personality trait, the two measures of tolerance (FID and VOD) need to be consistent within individuals and distinct between individuals across multiple contexts through time (*11*). To explore this, we exposed individual baboons to repeated trials in a range of environmental and social contexts (see contextual variables in Table 1) using two different observers varying in familiarity to the baboons. This setup allowed robust investigation into the tolerance personality trait hypothesis and generated two initial predictions: Environmental, social, methodological, and observer factors should play a minimal role in VOD and FID, i.e., individual baboon identity should account for a larger degree of the variance in FID and VOD (prediction 1), and individuals are consistent in both their FID and VOD responses to an approaching observer through time, i.e., individual repeatability (prediction 2). We explored the factors influencing VOD and FID separately using a Bayesian mixed-model approach. The final aspect of the tolerance personality hypothesis was to test for convergent validity [see (*24*)], through the correlation between displacement tolerance (derived from individual FID) and visual tolerance (derived from individual VOD). We predicted that visual tolerance and displacement tolerance would be correlated, such that both measure the same trait (prediction 3). This was explored using bivariate Bayesian mixed model analysis (*25*), retaining the predictors from the analyses for predictions 1 and 2.

**Table 1:** Factors hypothesized to influence VOD and FID in baboons. Contextual variables that could be major drivers of VOD and FID responses in habituated chacma baboons (examples from relevant literature supporting the inclusion of each hypothesis can be found in table S1).

**Factors**	**Link to sensory capacity/FID/personality**
**Response variable: VOD**	
Observer (pseudo-predator) identity, *X*_1_	Unfamiliar observer considered a greater threat, leading to increased risk perception and tendency to visually orient, resulting in longer VOD
Trial number, *X*_2_	(i) Increase or decrease in VOD with trial number indicative of habituation or sensitization (respectively) to FID approach methodology
	(ii) Consistent individual VOD response through time indicates personality trait.
Compatibility: Not engaged (looking/not looking), engaged (not looking), *X_3_*	Looking may enable animals to collect multiple types of information concurrently; in addition, being “not engaged” may afford focal animals a greater sensory capacity for detection. As a result, individuals looking as approach commences will visually orient toward approaching observer sooner resulting in longer VOD; engaged should yield shorter VOD.
Habitat (open/closed), *X*_4_	(i) “Open” habitats may afford individuals greater visibility, increasing likelihood of attending to approaching observer quicker, resulting in longer VOD.
	(ii) Open habitats are generally considered safer for baboons, as they permit earlier detection and avoidance of predators; therefore, risk perception could be lower, reducing tendency to visually orient toward approaching observer, resulting in shorter VOD.
	(iii) Open habitats may increase risk perception, as focal animals are less concealed from potential threats, increasing tendency to visually orient toward approaching observer, resulting in longer VOD.
	(iv) Open habitats have lower refuge availability, which may increase risk perception, resulting in longer VOD.
Height (ground/above ground), *X*_5_	“Above ground” may afford individuals greater visibility, resulting in longer VOD. In this context, above ground is <50 cm from ground level and is unlikely to qualify as potential refuge and therefore should not influence risk perception.
Number of neighbors within 5 m, *X*_6_	(i) As number of neighbors increase, the likelihood of a neighbor visually orienting toward the approacher increases, i.e., collective detection, which could result in longer VOD.
	(ii) As number of neighbors increase, the likelihood of predation decreases reducing risk perception and the tendency to visually orient toward the approach observer, resulting in shorter VOD.
	(iii) Increasing number of neighbors may mask both the visual and audible cues associated with the observer’s approach, resulting in shorter VOD, e.g., neighbors draw visual attention away from observer or noises from neighbors mask the sounds of observer’s footsteps during approach.
Neighbor flight, *X*_7_	Local conspecifics initiating flight before the focal animal will increase risk perception and evoke vigilance. Both factors could lead to focal animals visually orienting toward approaching observer sooner, resulting in longer VOD.
External factors (local alarms, aggressions within 5 min), *X*_8_	Localized threatening stimuli lead to increased risk perception and tendency to visually orient, resulting in longer VOD.
	Localized visual and audible stimuli may reallocate some of the focal animal’s finite attention, resulting in longer VOD.
	
**Response variable: FID**	
VODI, *X*_9_	When visual orientation interval (distance between VOD and FID) is long, focal animals will flee sooner, resulting in longer FID.
Engaged/Not engaged, *X*_10_	FID will be higher if focal animal was engaged at the start of the approach, as flight costs are higher because of interrupted social time (i.e., grooming) or loss of food patch (i.e., foraging).
Observer (pseudo-predator) identity, *X*_1_	Unfamiliar observer is considered a greater threat; therefore, FID should be greater for unfamiliar observer
Trial number, *X*_2_	(i) Increase or decrease in FID with trial number indicative of sensitization or habituation (respectively) to FID approach methodology
	(ii) Consistent FID response through time indicates personality trait.
Habitat (open/closed), *X*_4_	(i) Open habitats are generally considered safer for baboons, as they permit earlier detection and avoidance of predators; therefore, risk perception could be lower, resulting in shorter FID.
	(ii) Open habitats may increase risk perception, as focal animals are less concealed from potential threats, resulting in longer FID.
	(iii) Open habitats have lower refuge availability, which may increase risk perception, resulting in longer FID.
Number of neighbors within 5 m, *X*_6_	(i) Risk diluted with greater number of neighbors; therefore, FID should decrease as number of neighbors increases.
	(ii) Increasing number of neighbors increases localized visual and audible stimuli and therefore may reallocate some of the focal animal’s finite attention resulting in decreased FID.
Neighbor flight, *X*_7_	Local conspecifics initiating flight before the focal animal will increase risk perception and therefore increase FID.
External factors (local alarms, aggressions within 5 min), *X*_8_	(i) Localized threatening stimuli leads to increased risk perception and therefore increased FID.
	(ii) Localized visual and audible stimuli may reallocate some of the focal animal’s finite attention therefore decreasing FID.

## RESULTS

### Perceived threat level of approaching observers

We completed 1656 trials across 69 individual baboons (24 trials each; table S3), with behavioral responses recorded to understand the perceived threat level observers represented (Table 2). Observers do not appear to be considered equivalent to a predator. Instead, baboon responses mimic typical responses to approaches from dominant or threatening conspecifics. This suggests that observers are unlikely to be considered “neutral” but are instead more equivalent to a high-ranking social threat.

**Table 2:** Responses by baboons to approach and hypothesized meaning. Hypothesized individual baboon behavioral response to human approaches and the threat level these responses are considered equivalent to.

**Observer considered:**	**Equivalent to** **predator**	**Equivalent to social** **threat**	**Minimal threat**	**No threat**	**No. of observations** **(percentage of total** **observations)**
Response predictor					
Alarm bark	Y	–	–	–	0 (0%)
Flight direct to refuge (rocks, trees, or cliff)	Y	–	–	–	0 (0%)
Rapid flight/sprinting response	Y	Y	–	–	0 (0%)
Displacement with geck/ grimace	–	Y	–	–	16 (0.97%)
Animal passively displaces	–	Y	Y	–	1637 (98.85%)
Flinch/startled before flight*	–/*	–/*	–/*	–/*	3 (0.18%)
Animal is not displaced	–	–/*	–	Y	0 (0%)
Animal is not displaced and threatens observer	–	–	–	Y	0 (0%)

*Flinch or startled suggests that the focal animal detected observer within its usual tolerance level.

### VOD model

We implemented a maximal (or “global”) model containing all of our predictors of VOD with results suggesting that the compatibility, habitat, and number of neighbors variables were the most informative covariate predictors for VOD, with the envelope constraint well controlled for [Table 3: VOD delay (VODD) estimate = −0.02, Rhat = 1.00]. Compatibility variables seem to have a consistent influence on VOD, with longer VOD (earlier detection) for both looking and not engaged not looking categories compared to animals that were fully engaged, although the mean conditional effect estimates of engaged and looking differed by only 60 cm, suggesting that the detection capabilities of baboons may not be completely limited when not looking or when performing engaged behaviors. Animals in open habitats also detected observers sooner (longer VOD), although the effect was not as strong as the compatibility variables. Number of neighbors had a small negative estimate, but its credible intervals did not overlap zero, suggesting weak yet consistent effect. VOD was also longer (earlier orientation) when neighbors fled before the focal animal, although credible intervals included zero. The remaining covariates did not appear to add considerable explanatory power to predicting VOD, as each had estimates close to zero and credible intervals overlapping zero (see Table 3).

**Table 3:** VOD model summary. Parameter estimates for the model describing the relationship between VOD and the predictor variables. CI, credible interval.

**Population-level effects**							
	**Estimate**	**Est. error**	**1–95% CI**	**U-95% CI**	**Rhat**	**Bulk_ESS**	**Tail_ESS**
Intercept	1.06	0.08	0.9	1.22	1.00	23,289	35,333
VODD	−0.02	0.01	−0.03	0	1.00	34,760	41,624
Looking	0.21	0.02	0.17	0.25	1.00	69,337	46,658
Not engaged not looking	0.11	0.02	0.06	0.16	1.00	70,821	47,802
Open (Habitat)	0.15	0.02	0.12	0.19	1.00	73,748	46,813
Ground (Height)	0.06	0.05	−0.04	0.16	1.00	74,865	45,743
Number of neighbors	−0.05	0.01	−0.06	−0.03	1.00	76,910	46,466
Neighbor flee first	0.08	0.04	0	0.16	1.00	78,003	46,586
External factors within 5 min	0.02	0.03	−0.04	0.08	1.00	79,045	47,160
Unfamiliar observer (AB)	−0.04	0.07	−0.19	0.11	1.00	17,011	28,032
Trial number	−0.01	0.01	−0.02	0.01	1.00	18,351	29,277
Unfamiliar observer (AB): Trial number	0.01	0.01	−0.01	0.03	1.00	17,138	26,376
							
Family specific (log-normal)							
Sigma	0.31	0.01	0.3	0.32	1.00	48,397	43,998
							
Group-level effects							
Date (58 levels)							
sd(Intercept)	0.14	0.02	0.1	0.18	1.00	17,027	32,825
							
Individual identity (69 levels)							
sd(Intercept)	0.24	0.03	0.18	0.31	1.00	13,558	27,638
sd(VODD)	0.04	0.01	0.02	0.05	1.00	19,663	31,617
sd(ObserverAB)	0.09	0.04	0.01	0.17	1.00	7,956	13,090
sd(TrialNo)	0.01	0	0	0.02	1.00	7,995	11,891
sd(ObserverAB:TrialNo)	0.01	0.01	0	0.02	1.00	5,454	12,200
cor(Intercept,VODD)	0.57	0.19	0.15	0.89	1.00	14,071	24,184
cor(Intercept,ObserverAB)	0.21	0.31	−0.46	0.76	1.00	25,091	33,269
cor(VODD,ObserverAB)	0.16	0.33	−0.53	0.74	1.00	20,671	33,171
cor(Intercept,TrialNo)	−0.68	0.22	−0.94	−0.1	1.00	22,289	21,411
cor(VODD,TrialNo)	−0.3	0.29	−0.8	0.32	1.00	17,875	27,326
cor(ObserverAB,TrialNo)	−0.17	0.37	−0.81	0.57	1.00	17,569	30,321
cor(Intercept,ObserverAB:TrialNo)	0.35	0.3	−0.37	0.82	1.00	18,449	21,803
cor(VODD,ObserverAB:TrialNo)	0.29	0.31	−0.41	0.8	1.00	27,315	29,181
cor(ObserverAB,ObserverAB:TrialNo)	−0.05	0.39	−0.72	0.72	1.00	17,705	33,765
cor(TrialNo,ObserverAB:TrialNo)	−0.47	0.34	−0.91	0.4	1.00	9,755	21,676

### FID model

The maximal model for FID revealed that the engaged, habitat, and number of neighbors variables were the most informative covariate predictors for FID. The model results suggest that the envelope constraint was well controlled for [Table 4: VOD interval (VODI) estimate = −0.04, Rhat = 1.00] but provide little support for the Flee Early and Avoid the Rush (F.E.A.R.) hypothesis (*26*) (see Table 1: *X*_9_), as a minor negative estimate was produced for VODI with credible intervals both close to zero (Table 4). Animals that were not engaged at the start of an approach have longer FIDs (i.e., displace sooner), with animals in open habitats also appearing to displace earlier, resulting in longer FIDs. Animals that were on the ground throughout the approach had longer FIDs than animals slightly above the ground, although credible intervals included zero. Number of neighbors produced a consistent (narrow credible intervals) but weak negative effect. The effect of the unfamiliar observer produced a weak negative estimate, but credible intervals overlapped zero, suggesting little confidence in this factor being an important driver of FID. The remaining covariates did not appear to add considerable explanatory power to predicting FID, as each had estimates close to zero and credible intervals overlapping zero (see Table 4).

**Table 4:** FID model summary. Parameter estimates for the model describing the relationship between FID and the predictor variables.

**Population-level effects**							
	**Estimate**	**Est. Error**	**1–95% CI**	**U-95% CI**	**Rhat**	**Bulk_ESS**	**Tail_ESS**
Intercept	0.67	0.1	0.47	0.87	1.00	13,556	28,565
VODI	−0.04	0.01	−0.07	−0.01	1.00	45,436	45,243
Engaged	0.14	0.02	0.1	0.18	1.00	97,776	46,015
Open (Habitat)	0.12	0.02	0.08	0.16	1.00	91,775	47,949
Ground (Height)	0.12	0.06	0	0.23	1.00	100,351	48,107
Number of neighbors	−0.08	0.01	−0.09	−0.06	1.00	98,909	47,398
Neighbor flee first	0	0.05	−0.09	0.09	1.00	94,500	45,544
External factors within 5 min	0.01	0.04	−0.06	0.08	1.00	94,487	45,998
Unfamiliar observer (AB)	−0.14	0.08	−0.3	0.03	1.00	19,463	30,667
Trial number	−0.02	0.01	−0.04	−0.01	1.00	21,542	34,353
Unfamiliar observer (AB): Trial number	0.02	0.01	0	0.05	1.00	17,996	27,736
							
Family specific (log-normal)							
Sigma	0.36	0.01	0.34	0.37	1.00	55,469	45,557
							
Group-level effects							
Date (58 levels)							
sd(Intercept)	0.14	0.02	0.11	0.19	1.00	17,300	31,725
							
Individual identity (69 levels)							
sd(Intercept)	0.49	0.05	0.4	0.6	1.00	13,780	25,841
sd(VODI)	0.06	0.02	0.02	0.09	1.00	10,338	13,826
sd(ObserverAB)	0.18	0.04	0.1	0.26	1.00	17,276	16,843
sd(TrialNo)	0.01	0	0	0.02	1.00	11,643	13,855
sd(ObserverAB:TrialNo)	0.01	0.01	0	0.02	1.00	8,880	18,037
cor(Intercept,VODI)	0.26	0.22	−0.16	0.7	1.00	22,518	25,743
cor(Intercept,ObserverAB)	0.04	0.2	−0.33	0.44	1.00	34,506	35,920
cor(VODI,ObserverAB)	0.16	0.28	−0.39	0.68	1.00	10,048	18,984
cor(Intercept,TrialNo)	−0.46	0.25	−0.84	0.15	1.00	43,028	29,416
cor(VODI,TrialNo)	−0.25	0.33	−0.81	0.45	1.00	17,519	28,304
cor(ObserverAB,TrialNo)	−0.39	0.29	−0.86	0.26	1.00	21,982	31,250
cor(Intercept,ObserverAB:TrialNo)	−0.12	0.33	−0.73	0.56	1.00	45,303	39,936
cor(VODI,ObserverAB:TrialNo)	−0.36	0.35	−0.88	0.47	1.00	26,569	34,646
cor(ObserverAB,ObserverAB:TrialNo)	−0.05	0.37	−0.7	0.69	1.00	31,439	41,766
cor(TrialNo,ObserverAB:TrialNo)	−0.13	0.39	−0.77	0.67	1.00	19,037	35,675

**Fig. 2:**
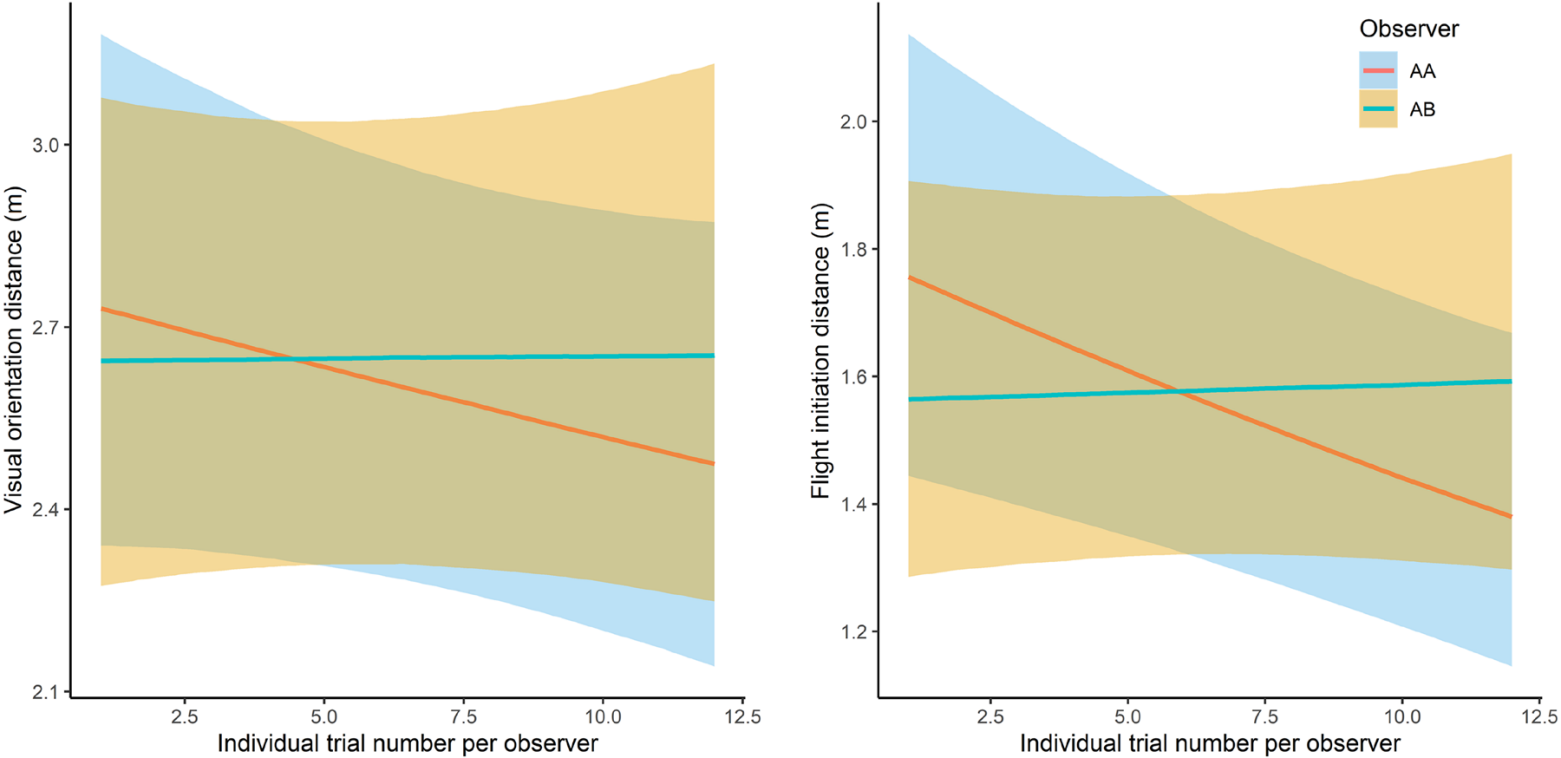
Conditional effect plots for interaction between observer identity and individual trial number per observer from VOD and FID models. The plot represents conditional predictions of the regression curve when all fixed effects are held constant apart from the interaction (observer × individual trial number per observer); the mean was used as the measure of central tendency, with the shaded areas displaying the relevant credible intervals (2.5 and 97.5% percent quantiles). AA represents the familiar observer, and AB represents the unfamiliar observer.

These results mimic those found for VOD, with little suggestion that habituation/sensitization to methodological stimulus took place. In addition, little difference was found between observers (for VOD or FID), both in absolute terms and in their individual effect over the course of successive trials (see Tables 3 and 4 and Fig. 2). For both FID and VOD, the “unfamiliar” observer produced consistent estimates through successive trials. The baboons’ responses to a “familiar” observer (AA) produced a declining trend for both VOD and FID, suggesting that study animals were initially sensitive to the approaches of AA but slightly habituated over the course of successive trials (Fig. 2); however, the effect did not carry sufficient statistical weight. The study group’s prior experience of being observed by AA may suggest that actions of an observer outside of their “normal” behavior (i.e., the repeated direct FID approaches) were considered somewhat threatening to baboons, but the declining trend also suggests that the study group as a whole adapted and habituated to this unusual behavior quite quickly.

### Tolerance as a personality trait

To test whether visual tolerance (VOD) and displacement tolerance (FID) were distinct among individuals, we removed the individual identity random effect from each model and used log-score stacking [see (*27*)] to combine its Bayesian predictive distribution with the same model inclusive of individual identity. For both the VOD and FID, removal of the individual identity random effect resulted in a less informative model with log-score stacking favoring the inclusion of individual identity in both cases: VOD model with individual identity weight, 0.987; model without individual identity weight, 0.013; FID model with individual identity weight, 0.999; and model without individual identity weight, 0.001. In addition, parameter estimates and credible intervals from each model suggested that individual identity was a key predictor of VOD and FID [see sd (intercept) in Tables 3 and 4]. In both cases, estimates for ID were greater than each of the covariate predictors, while credible interval did not overlap zero. These results strongly suggest that individual identity was the most important driver of VOD and FID, emphasizing that both measures are distinct among individuals.

To test whether VOD and FID were consistent within individuals, we calculated the intraclass correlation coefficient (ICC) from the univariate VOD and FID models using an enhanced agreement repeatability protocol (see the “Statistical analysis” section for description) (*28*). We observed moderate ICC estimates for individual identity in both VOD (individual identity ICC, 0.38; highest density intervals (HDI) for posterior samples at 95% intervals, 0.24, 0.51) and FID (individual identity ICC, 0.65; HDI, 0.56, 0.74) after accounting for variance explained by fixed effects and observation date. Following Houslay and Wilson (*25*), we used the above protocol to also derive ICC calculations from a bivariate model (see below) for both VOD (ICC, 0.38; HDI, 0.27, 0.50) and FID (ICC, 0.62; HDI, 0.54, 0.74), each producing almost identical values to the univariate approach. In each case, the lower bound of the Bayesian 95% credible interval was not close to zero, indicating that there is at least moderate confidence in a nonzero proportion of phenotypic variance in both VOD and FID being explained by within-individual consistency (*25*). While these ICC estimates may be considered “moderate” (*29*), personality analyses have previously interpreted values as low as 0.168 to be suggestive of repeatability (*23*), with 0.342 reflecting repeatability in male reindeer (*30*), suggesting that both the visual and displacement responses of this baboon group have a clear personality component. These findings are strong evidence that both tolerance behaviors were consistent within, and distinct among individuals, and were therefore taken to indicate that the behaviors manifest themselves as a personality trait (*31*).

### Convergent validity

We followed (*25*) and implemented a bivariate Bayesian model to assess convergent validity, with VOD and FID included as response variables and each predicted by the same covariates used in the maximal models. We used the same priors, random structure, and log-normal response distribution, as used in univariate models. After fitting this model, we estimated the mean and credible intervals of the correlation between VOD and FID from the bivariate model covariances. A new posterior distribution was constructed from the among-individual correlation by dividing the covariance between VOD and FID by the product of the square root of their individual trait variances, thus standardizing their covariances on a scale from −1 to 1. This process produced a mean correlation of 0.875 between visual tolerance (VOD) and displacement tolerance (FID), with a lower high-density credible interval of 0.767 and a higher HDI of 0.967, suggesting a very high degree of confidence in concluding a statistically significant correlation and thus meeting the requirement for convergent validity (*25*).

**Fig. 3:**
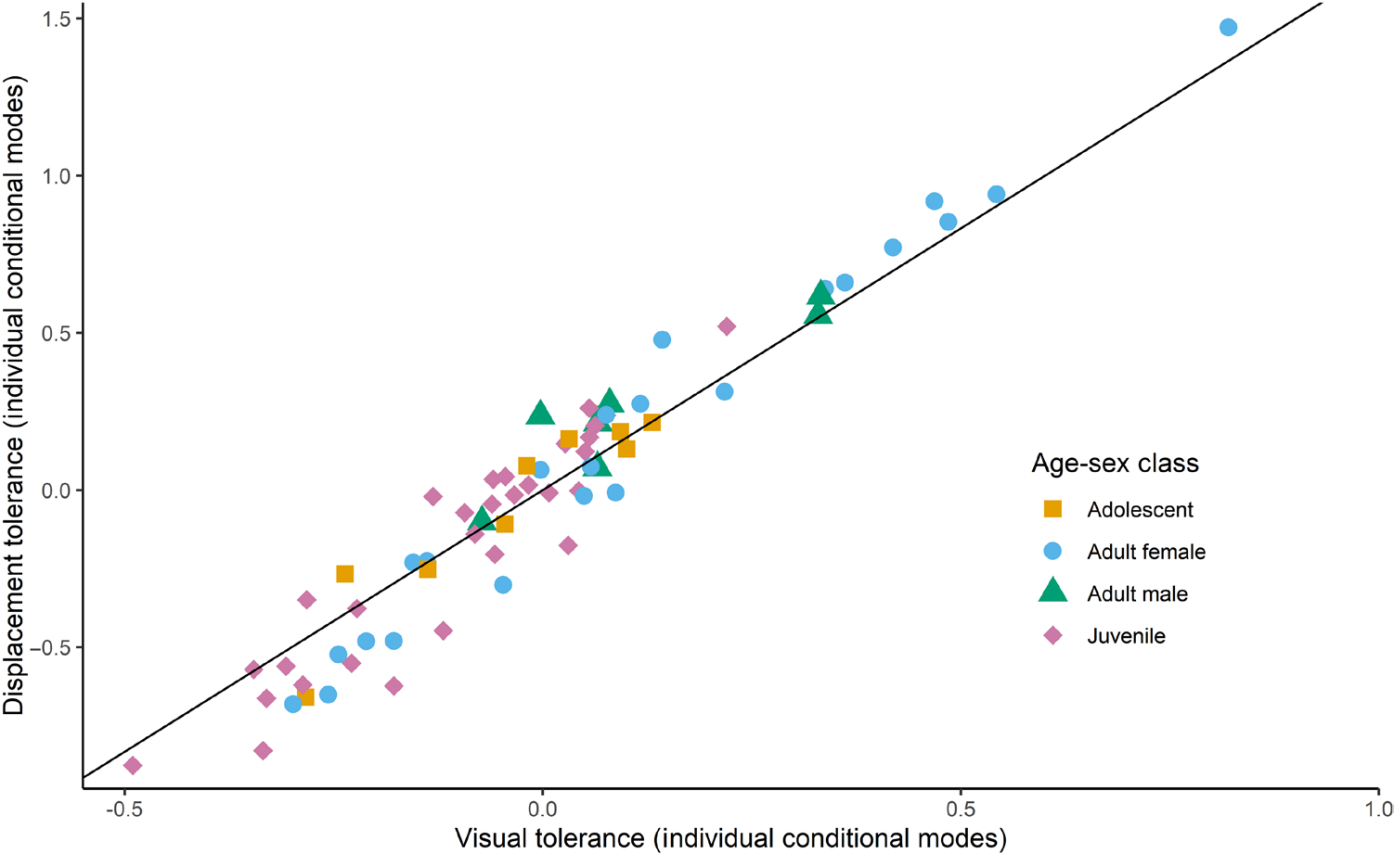
Convergent validity regression. Regression relationship between visual tolerance and displacement tolerance. Estimates were derived from bivariate Bayesian model; lower values indicate greater “tolerance.” Each point represents the conditional modes of an individual baboon (*n* = 69) for each tolerance trait.

We additionally extracted the conditional modes (posterior modes) of each individual baboon for both VOD and FID (see Fig. 3). Conditional modes are the equivalent of best linear unbiased predictors, which have been used elsewhere in personality research [e.g., (*22*, *25*)]. Conditional modes terminology reflects the fact that the computation works to maximize the density of the individual identity random effect conditioned on the variance-covariance matrix of the fitted model framework and observed data (*32*). The individual conditional modes occupied a range of correlated tolerance estimates across the spectrum (see Fig. 3), with all age-sex classes having individuals spread across large parts of the spectrum. A small number of individuals (seven adult females, two adult males, and one juvenile) seem particularly sensitive to approaches by observers (high values on both axes), while two adult females, one adolescent, and six juveniles appear exceptionally tolerant of approaches by observers.

## DISCUSSION

Our findings indicate that the behavioral responses of baboons to approaches by human observers were most consistent with responses toward high-ranking social threats (passive displacement), although active responses were also recorded on rare occasions. This suggests that human observers are not neutral to habituated primates. We failed to detect any evidence that a suite of environmental (height), social (neighbor flight and external events), and methodological/observer variables (observer identity and trial number) influenced VOD or FID. Although some factors (such as baboon behavior at that start of approach, habitat, and number of neighbors) played a role in how quickly baboons visually oriented toward or were displaced away from an approaching observer, these factors were largely overshadowed by the role of individual identity in predicting when visual orientation or displacement would occur. Bayesian stacking weights [see (*27*)] strongly favored the models inclusive of individual identity, suggesting that VOD and FID responses were distinct among individuals, providing support for prediction 1: Individual baboon identity should account for a larger degree of the variance in VOD and FID than other variables. In addition, ICC estimates revealed that VOD and FID were consistent within individuals (i.e., repeatability), supporting prediction 2: within-individual consistency in VOD and FID responses. Last, we found a strong correlation between visual and displacement tolerance, supporting prediction 3: VOD and FID are different measures of the same personality trait, i.e., convergent validity. Together, these results suggest that two implicit assumptions concerning habituation in wild animals are not applicable to this group: first, that observers were considered neutral and, second, that habituation (i.e., tolerance) was equal across study animals.

Research into animal personalities has grown expansively, exploring traits such as aggressiveness, shyness/boldness, avoidance of novelty, exploration and activity, and sociality (*18*, *33*). Our study is the first to show empirically that tolerance toward human observers is a personality trait in wild, habituated animals. Focus on individual animals is somewhat lacking in FID literature thus far, with individual repeatability often assumed (*34*). Our approach illustrates that extensive, robust sample sizes can be obtained for animals in the wild and so provide a framework for future research. While these findings could be considered a single-group specific phenomenon, the implications of such a personality trait has important implications for primate behavioral ecology and animal behavior studies in general (as discussed below) and so is in need of further research in other contexts. Our framework for recording the behavioral response of animals as a result of each approach (see Table 2) is relatively simple to design and implement and therefore should be easy for future behavioral studies to incorporate. The approach should also be an important precursor to any FID research, as it is often assumed that humans are considered equivalent to predators (*35*). This assumption was not supported in this study and may become questionable as wild populations become increasingly exposed to anthropogenic disturbance (*36*).

We found an exceptionally strong correlation between individual displacement and visual tolerance traits, a result we interpret as a true biological effect. We did not detect any evidence of a heteroskedastic relationship throughout the convergent validity analyses (see Fig. 3), suggesting that individuals have similar levels of visual and displacement tolerance. To validate this result and control for alternative explanations, we subset our data into two evenly sampled and independent datasets; the VOD dataset used even trial numbers (trial numbers 2, 4, 6, etc.), while the FID dataset used odd trial numbers. We ran the same bivariate Bayesian mixed model, ICC, and convergent validity analyses, with almost identical results (see table S2). There is thus a high degree of confidence in the results and issues relating to the envelope constraint found in typical FID analyses [see (*37*)] that were not driving this correlation, and neither were problems relating to shared method variance [see (*24*)]. As a result, there are clear biological grounds for suggesting convergent validity between these factors.

One criticism of our methodological approach may be that direct approaches toward focal animals do not mimic the intent of observers when collecting behavioral observations on wild animals (i.e., observers are normally attempting to be neutral). Nevertheless, incidental displacements inevitably occur throughout the observation process; whether wild animals detect a difference between “accidental” and “deliberate” approaches is up for debate. Since we found consistent visual orientation and flight responses across a range of start distances (SDs) (range, 2.5 to 33.8 m; table S3), it seems unlikely that “sensitive” individuals in the study group only visually orient toward observers when they consider them to be directly approaching them. One methodological adaption may be to use approaches that move parallel to the focal animal or are designed to pass by the animal at predetermined distances; however, this approach would likely not produce a distance measure for all individuals as tolerant phenotypes may not respond to approaches in any detectable way. Therefore, direct approaches (on habituated animals) are likely required if research is focused on measuring VOD and FID within a personality paradigm. However, specifying a common SD and varying approach angles systematically would be an interesting area for future research to explore. This could be particularly enlightening for understanding other questions, such as how the number of observers concurrently observing habituated animals could influence a group’s activity budgets, e.g., increasing the number of observers increases the number of visual stimuli that “intolerant” individuals need to attend to (visual) or avoid (displacement).

Alongside the personality findings, we found that baboons using any form of “looking” behavior appeared able to detect approaching observers quickest; however, the mean conditional effect difference between “engaged” and looking was 57 cm, suggesting that baboons have the sensory capacity to detect local threats regardless of their behavior. This is an interesting result when applied to the typical notions of a foraging-vigilance trade-off found throughout vigilance research on several taxa [e.g., (*38*–*40*)]. Do baboons need to be vigilant to detect threats? Active engagement in foraging or grooming appeared to hinder “detection” at only a very minor level, especially when considering that SDs for approaches covered a broad range of distances (range, 2.5 to 33.8 m). These results suggest that baboons do not need to be “vigilant” for approaching threats to detect them. It could be possible that during engaged behaviors, baboons are able to switch to using auditory cues to monitor the position of approaching threats. The result that VOD decreased (detection time increased) as number of neighbors increased may support this notion, as additional conspecifics may mask the audible cues associated with the approaching observer (see table S1). Although more work is required in this area, our results suggest that baboons may be able to collect multiple types of information concurrently, especially when stimuli are local. This finding is valuable for primate vigilance research, as compatibility between general looking/scanning behaviors and threat detection has proved a popular assumption in many studies to date (*41*).

The assumption that observers play little role in the behaviors that they record is implicit throughout animal behavior research, with habituation assumed to lead to study animals “ignoring” human observers or that observer presence is rarely disruptive [e.g., (*4*, *42*, *43*)]. Our results challenge this assumption, but the methodological and statistical approaches that we used offer future researchers a number of tools for ameliorating these observer-related issues. The framework can be used to produce visual and displacement tolerance estimates (i.e., conditional modes) for each individual animal. These variables, known as best linear unbiased predictors elsewhere [see (*25*)], could be used as predictor covariates when addressing a range of questions relating to observer affects. Researchers exploring questions relating to individual vigilance or looking patterns may need to control or investigate the role of tolerance in the behaviors that they record (*5*). Factors such as spatial cohesion and position could be partially determined by observer-governed phenotypic assortment, e.g., intolerant individuals occupying spatial positions that reduce the likelihood of being in proximity to observers (*44*). This would lead to biased recording of social networks, but the methods here could help identify these constraints. It has been argued that predation risk is not uniform across a group (*45*), and the methods here could be used to explore the interaction between individual-level tolerance traits and the typical spatial cohesion and positional patterns used by group members to avoid predation; do intolerant phenotypes weigh-up the risk of remaining on the periphery of groups with the risk of being in proximity to observers? Conversely, the human shield effect (*46*), where wild animals may perceive reduced risk of predation when human observers are present, may only be realized by animals with highly tolerant phenotypes. These tolerant phenotypes may also be able to exploit the tolerance differential and use observers as social tools to aid in accessing/retaining food patches or avoiding aggression; individual conditional modes would be a powerful asset for testing these effects.

Our analytical frameworks could be used to inform researchers of appropriate observation distances to sample animals from to reduce the chance of recording biased social network information and to minimize the effect that observers have on spatial cohesion and individual positioning with the group. For example, our study suggests that an observer distance of greater than 17 m would likely be required if the goal is to completely eradicate any localized observer effects (max VOD recoding, 16.7 m; max FID, 15.6 m). A distance of 5 m would still promote visual monitoring in approximately 10% of the group, i.e., seven individuals had average VODs of greater than 5 m, suggesting that while distance protocols could ameliorate observer effects in many instances, it is unlikely that observers could collect detailed observational data while maintaining distances unlikely to affect a focal animal and its neighbors. If future research attempts to conduct similar work, we highlight that our sampling effort was far greater than necessary (24 trials across each individual). When testing the even- and odd-numbered trials separately (as described above), we were able to fit models with 12 trials without convergence warnings or divergent transitions. This suggests that sampling efforts of six to eight trials per individual may be adequate and should make this process achievable in most contexts; hence, individual measures can be captured relatively quickly as a baseline before observation work commences.

We hope that this work can reignite a wider debate concerning the methodological and ethical assumptions relating to undertaking behavioral observations on wild animals in situ, not only in wild primates but also in other species, e.g., mongooses (*4*, *43*) and meerkats (*42*) where direct observations of study subjects is regularly used in research. Our results suggest that human observers are not neutral and that tolerance is not equal across the individuals within our group of habituated chacma baboons. It is unknown to what extent similar factors are at play in the host of other systems monitored by behavioral ecologists around the globe, but there is a need to investigate these factors to ensure that we are not systematically biasing results through our methodological choices.

## MATERIALS AND METHODS

### Ethics

This research was undertaken under ZA/LP/81996 research permit, with ethical approval from the Animal Welfare Ethical Review Board at Durham University.

### Study area

FID approaches were conducted in the field at the Lajuma Research Centre, western Soutpansberg Mountains, South Africa (central coordinates 29.44031°S, 23.02217°E) between October 2017 and April 2018. The altitude of this study area varies from 750 m above sea level to 1748 m at the peak of the mountain range (within the study area). The mountainous environment includes a complex mosaic of natural habitat types that belong to the Afromontane mist-belt communities, with natural habitats varying greatly in their structural characteristics (*47*). Although the majority of the land within the study site is classified as a private nature reserve, ecotourism takes place in these areas, while monoculture crop farming and livestock farming take place locally. These farming practices overlap with the core part of the study baboon group’s typical home range, with baboons regularly scared away from farm areas by workers clapping, yelling, or throwing stones; however, serious or fatal conflict has never been observed. The habituated study group appeared to differentiate between observers and farm workers, e.g., baboons will alarm in response to worker presence while concurrently allowing close observer proximity.

### Study group

The group was habituated circa 2005 and was the focus of intermittent research until the start of this study [see (*47*)]. Since 2014, the group has received consistent observational research in the form of full-day follows 3 to 4 days a week, with occasional gaps of up to a duration of 5 weeks. The group was typically followed from dawn to dusk on a 4 days on–3 days off schedule designed to maintain as much of their natural interactions with predators as possible. The study group contained between 76 and 85 individuals over the course of the study. One confirmed predation of an adult male baboon by an adult male leopard took place, while several other disappearances occurred, although the causes were unconfirmed. A total of 69 individuals were used in the final analyses: 21 adult females, 7 adult males, 4 adolescent males, 7 adolescent females, 13 juvenile females, and 21 juvenile males. Across the study period, several individuals changed age-sex class category (see text S1 for descriptions); as a result, the total number of individuals sampled does not equal the cumulative total for each age-sex class.

### FID approach procedure

When a focal animal was encountered, the observer moved to an appropriate distance (based on the distribution of previous approaches) and angle relative to the focal animal. This position had to be within a 90° field of view of the front of the focal animal’s head (45° either side of center), i.e., the focal animal’s head had to be broadly facing forward. Approaching from outside of this angle may have been challenging for baboons to detect approaching observers visually, forcing focal animals to rely on other cues instead. As baboons can rotate their heads quite far, this was more appropriate than using orientation of the focal animal’s body to judge start position. The approaching observer would wait for at least 10 s at the start position before commencing an approach and would only start an approach if there was no obvious response from the focal animal within this time. Trials were abandoned after 30 s if an approach was not started, such as where another baboon sat between observer and focal animal before starting the approach, the focal animal turned its head so that we could no longer approach directly within their visual field, or the focal animal was already looking toward the observer. In all scenarios, another focal animal was selected instead.

When ready to start the approach, we dropped a marker (a blue and purple spray-painted rock approximately 2 cm in diameter) behind our feet (to mark the SD). In all approaches, observers walked directly to the focal animal’s start position, without pausing at any point (*19*). During the approach, we dropped additional stones behind our legs to mark VOD, neighbor flight distance (i.e., neighbor within 5 m at start of approach is displaced before focal animal), and FID (i.e., the distance at which the animal moved away from its original position as a result of the approach). VOD was operationally defined as the focal animal directing their line of vision toward the face of the approaching observer, a behavioral marker shown during pilot work to be associated with detection of our approach. There were no instances of additional baboons interrupting the approaches or moving in between the focal animal and the approaching observer; however, approaches would have been abandoned in such circumstances.

Stones consistently landed in accurate locations, but a second observer was always present several meters behind the start position to confirm the location of the stones was accurate or subsequently adjust the position of stones that bounced into inaccurate positions. As each observer dropped the markers behind their legs during the approach, the sound of the small stone landing was apparently either masked by the observer’s footsteps (and other local noises) or was not a sufficient stimulus to warrant a visual orientation or flight. We did not observe any focal animal respond to the markers landing; however, approaches would have been abandoned if this had happened. We repeated three approaches when juvenile baboons picked up the stones. Distances between markers and the start position of the focal animal were then measured using a laser range finder (Leica DISTO DXT) and recorded on an electronic device (Samsung Galaxy J5, Samsung Town, Seoul, Republic of Korea), using a personalized application built with the software CyberTracker v3.466 (CyberTracker Conservation, Bellville, South Africa; http://www.cybertracker.org). After the approach was successfully completed, we noted the behavioral response of the focal animal (behaviors listed in Table 2). We excluded the behavioral marker “tail up,” as this can be hard to identify because of individual tail use varying.

### Sampling design and justification

To produce an equal sampling effort across the study group, each individual study animal was subjected to 12 approaches by two observers (24 in total). One observer was considered familiar (AA, had followed the group for approximately 3 years), and the second observer was considered unfamiliar (AB, conducted first FID approach on the first day with study group); the unfamiliar observer was always in proximity to the familiar observer, which may have diminished the initial novelty/threat perceived by the study group (toward the unfamiliar observer). The presence of the familiar observer was essential, however, to ensure accurate identification of study animals.

To confirm that both observers were making identical judgements for VOD and FID, we undertook 60 pilot trial approaches (30 for each observer). For each approach, one observer would drop the markers for VOD and FID, and the second observer would observe the approach and note whether they agreed with where markers were dropped on the basis of the focal animal’s behavioral responses. Both observers were in agreement for all distances throughout pilot work, i.e., the second observer did not disagree with the placement of the stones for either observer for any trial, suggesting a robust definition framework.

To control for time of day, each day was split into four time periods that were adjusted seasonally to ensure that each accounted for 25% of the current day length. We recorded six samples in each time period, three by each observer. Since certain intolerant animals were harder to sample (i.e., would displace before allowing FID approaches), focal individuals were selected pseudorandomly but sampled evenly across each time period. On average, 29 approaches were completed each day (min = 1 and max = 83) across 58 sampling days. We limited each individual to a maximum of two approaches within a single day.

The effect of SD has received a great deal of attention and is one of the strongest and most widely reported effects in FID literature [see (*48*)]. SD is determined by the observer, with recent best practice recommendations suggesting that researchers should systematically vary this distance to get a true understanding of the dynamics of escape behaviors (*19*). We attempted to distribute SDs evenly from close (approximately 3 m) to distant (8 m and beyond) for each individual (see table S3). These distances reflect the normal range of distances used when collecting behavioral data on the study group. Although most individuals received approaches across an even distribution of SDs, certain individuals did not permit close SDs (see table S3).

We controlled for approach speed by using a controlled walking pace during observational data collection. Both observers measured their walking speeds before study (20 trials walking between 5-m markers) and aligned their walking speeds to one another, resulting in almost identical walking speeds when tested again (20 observations each; AA: mean = 0.84 m/s, min = 0.74, and max = 0.95; AB: mean = 0.81 m/s, min = 0.76, and max = 0.90). When approaching, we focused our gaze on the focal animal’s forehead to maintain the same speed and posture throughout the approach (*49*). In addition, it allowed both observers to easily identify each parameter (visual orientation, neighbor movement, and flight). Direct eye contact was avoided, as this can startle baboons and is similar to their natural dominance behaviors.

### Contextual variables

As baboons can change behavior rapidly, we made no attempt to restrict approaches to certain behaviors; instead, we used an instantaneous scan sampling method to record contextual variables at the instant we commenced an approach. We recorded the following factors (see Table 1): whether the focal animal was performing engaged (foraging, giving grooming, and autogrooming) or non-engaged (resting, receiving grooming, and chewing food) behaviors, looking [see (*41*)] or not looking, whether the animal was on the ground/aboveground, current habitat type (open/closed), number of neighbors within 5 m of the focal animal, and alarms or aggressions within 5 min before approach. During the process, we noted the trial number that each individual baboon had received per observer, i.e., separate trial number scores of 1 to 12 for each observer.

We made approaches in all the habitats that the baboons use (see text S2 for descriptions) but did not undertake approaches where individuals were adjacent to large rocks or cliff edges, as these limit escape options. Approaches were only made when there were no obstructions between the focal baboon and the approaching observer, allowing consistent posture and head and eye direction. We did not systematically vary the habitat that we undertook approaches for each individual; as a result, certain individuals may have received approaches in some habitats more than others. We did make approaches toward individuals sat on small rocks or low-hanging branches within 0.5 m off the ground; this was recorded as a categorical variable (above ground/on ground). We did not attempt approaches on individuals higher than this, as the approach would no longer be direct, as the observer could not directly walk through the target animal’s start location. Alarms or aggressions could be from any individual within the group and were simply used as a proxy for increased risk perception. Last, we chose 5 m as the distance for recording number of neighboring conspecifics, as this was a well-practiced measurement consistently undertaken during previous research on this baboon group by AA and reflects a compromise between maximizing information in high-visibility areas and minimizing error in low-visibility habitats.

### Statistical analysis

#### *Drivers of VOD and FID*

We used complete (i.e., maximal/global) models for both VOD and FID analyses (inclusive of all contextual effects; Table 1). The VOD and FID models included VODD and VODI, respectively, as covariates to control for envelope constraint (see Materials and Methods). The only interaction term included was the interaction of observer identity and trial number, as this explored whether any habituation/sensitization effects took place for each observer through time while investigating the separate effects of observer identity and trial number on both VOD and FID. We fitted both models with random intercepts over date and individual identity. We additionally specified a random slope for the interaction between observer identity and trial number over individual identity, allowing the rate at which individuals habituate/sensitize to each observer to vary between individuals. All varying effects of individual identity were modeled as correlated. The inclusion of individual identity was validated using log-score stacking to combine Bayesian predictive distributions, which is recommended in an “M-open” situation (*27*).

The “compatibility” variable only appeared in the VOD model as, theoretically, the distinction between looking, engaged, and “not looking not engaged” should only influence the VOD response variable. The variable “engaged/not engaged” was only included for FID as a measure of the focal animal having costs associated with early departure, i.e., loss of social time or foraging patch. The variables of habitat (open/closed), number of neighbors, neighbor flight, and aggressions/alarms within 5 min of the start of the approach were considered for both VOD and FID. We did not explore the factors determining why a focal animal may have been looking at the start of an approach, but factors such as number of neighbors and aggressions/alarms within 5 min could alter risk perception and subsequently prime an individual to respond faster to an approaching threat, e.g., increasing neighbors could decrease risk perception, resulting in slower visual orientation and shorter VOD; the opposite would potentially be true for an alarm or aggression within 5 min, i.e., increased risk perception leading to individuals having a faster tendency to visually orient, thus producing a longer VOD. The height variable (ground/above ground) was included within for VOD as “above ground” had a maximum of 50 cm and therefore should not alter risk perception but could allow for earlier detection of threats, i.e., increased VOD.

All models were fit using the brm function from the brms package (*50*) in the R software (*51*). The brm function commands samples to be drawn from the posterior distribution via the package Rstan (*52*), which interfaces with the probabilistic programming language Stan via the C++ toolchain in Rtools (*53*). The brm function implements Hamiltonian Monte Carlo in combination with the No-U-Turn Sampler extension. For each model, we ran six Hamiltonian Markov chains for 15,000 iterations (including 5000 warmup iterations) with adapt delta set to 0.95, to provide algorithms that converge efficiently for multilevel models (*50*). The Gelman-Rubin convergence diagnostic [Rhat (*54*)] was used to assess Markov chain Monte Carlo convergences by comparing the estimated within- and between-chain variances of each factor within the model. All models had Rhat of 1.00 for all factors, suggesting very accurate estimates of the posterior distribution (*50*). Normal priors (mean = 0, standard deviation = 100) were assigned for fixed effects within the brm function; the random effects were assigned default half Student *t* priors (df = 3, mean = 0, standard deviation = 10).

All models were fit with log-normal response distributions (family) and default link functions (*50*). Log-normal was initially decided after visual assessment of the response distribution using Cullen and Frey plots (descdist function) and further assessed using the qqcomp function. We subsequently validated this in all candidate models by checking the residual standard deviation of each model, with Rhat = 1.00 in all cases, indicating accuracy of the response variables with regard to the log-normal response distribution, i.e., the standard deviation of VOD/FID points formed around the log-normal functions was minimal. The random structure of all models included fitting the random structure of observation date crossed with individual identity, which was paramount to our personality and habituation/sensitization hypotheses. All models included the intercept; forcing the SD × FID or SD × VOD regressions through the origin has been subject to debate (*55*); however, as we did not start approaches if focal animals were already looking at us and, therefore, SD > VOD, we assumed that the predicted relationship of SD with FID/VOD changes with increasing SD applied and therefore followed the advice in (*55*) and similar work by other authors [e.g., (*22*)].

In recent literature, the FID ≤ alert distance/VOD ≤ SD relationship has been referred to as a “constrained envelope” and results in some underlying issues with analysis due to extreme heteroscedasticity breaking model analysis assumptions. Although other approaches have been suggested, e.g., quantile regression (*56*) and Phi index (*36*), we elected to control for varying SD indirectly by including one of the other independent distance measures, i.e., to standardize the analysis for variance in SD, we included VODD (as a covariate and as a random slope over individual identity) in all models analyzing VOD and VODI (as a covariate and as a random slope over individual identity) in all models analyzing FID (*57*). This allowed us to retain the covariate predictor variables in the analysis, which would not be possible with the Phi index. Quantile regression was not considered, as individual sample sizes were not above the minimum threshold of 50. We discounted a final option of ignoring the intercept of the relationship [see (*48*)], as this has previously been criticized (*36*).

#### *Tolerance as a personality trait: Visual tolerance and displacement tolerance*

We tested the personality hypotheses for prediction 1 (VOD and FID distinct among individuals) by comparing Bayesian stacking weights for each maximal model with and without the individual identity random effect. To achieve this, we firstly estimated the pointwise out-of-sample prediction accuracy from each maximal model (VOD and FID) inclusive and exclusive of the individual identity random effect using leave-one-out cross-validation (LOO) from the “loo” package (*58*). loo uses a Pareto smoothed importance sampling (PSIS) procedure for regularizing importance weights when computing LOO (hereafter termed as PSIS-LOO) (*27*). We found good PSIS approximation reliability by inspecting the estimated shape parameter $\hat{k}$ diagnostic values in the generalized Pareto distribution; for all models, we had no left-out data points for which $\hat{k}$ > 0.7 (*27*, *59*). Bayesian stacking was undertaken using the “stacking_weights” function from the loo package. Each maximal model was compared to the same maximal model, excluding individual identity using log-score stacking to combine Bayesian predictive distributions. When comparing two models, if the one model does consistently better than the other model at every pair of data points, then the stacking weight is equal to 1 (*59*). Thus, a stacking weight of 1 signifies that one model has predicted every data point better than the other model and offers substantial predictive power over the other model. Personality research has previously used likelihood ratio tests to test statistical significance of repeatability of linear mixed-effects models with and without identity effects (*23*); however, this Bayesian stacking approach has been strongly recommended within a Bayesian framework and highlights the extent to which individual identity accounts for variance within the maximal models (*27*).

We calculated the ICC, otherwise termed repeatability, to assess personality hypothesis prediction 2 (within-individual consistency in VOD and FID). ICC is typically estimated as the ratio of the variance associated with the individual identity effect divided by the total variance, i.e., sum of individual and residual variances, (VAR_ind_/VAR_ind_ + VAR_resid_), with ICC informing researchers of the degree of variance explained by individual differences, and thus is a measure of individual consistency (*22*). To achieve this, we extracted the relevant variance components from the maximal VOD and FID (univariate) models using the “VarCorr” function, squaring the estimated standard deviations to produce estimated variance values, and used these values to create two new posterior distributions for VOD and FID separately (*25*). The ICC value calculated for individual identity represents the ratio between (i) the variance explained by drawing from the posterior predictive distribution not conditioned on individual identity or observation date and (ii) the variance explained by posterior predictive distribution conditioned on individual identity (with random slopes for VODD/VODI, and the interaction between observer identity and trial number) crossed with observation date (separately). The calculated ICC values (one for each model, VOD and FID) for individual identity controls for the variance explained by observation date and also by the wider fixed effects structure by drawing from the posterior predictive distribution in each calculation phase, therefore producing values equivalent to enhanced agreement repeatabilities (*28*).

To investigate whether visual tolerance (VOD) correlated with displacement tolerance (FID), i.e., convergent validity (*24*) (prediction 3), we used a bivariate Bayesian mixed-effects model fitted with VOD and FID as response variables. The model was fit with the same fixed effects structure from each response variable’s maximal model, log-normal response distributions, and the same priors as described for the univariate model analyses. After fitting this model, we extracted the variance components from the model using the VarCorr function, again squaring the relevant standard deviations to calculate estimated variance values. This allowed us to create a posterior distribution of the among-individual correlation by dividing the corresponding variance between VOD and FID by the product of the square root of their individual trait variances, which standardizes their covariances to a scale from −1 to 1.

#### *R code*

Univariate maximal VOD model: brm (VOD ~ VODD + Compatibility + Habitat + Height + Number of neighbors + Nearest neighbor flee first + External events within 5 min + Observer*Trial number + (1|Date) + (1 + VODD + Observer*Trial number|p|ID), data = FID, family = log-normal, prior = prior, chains = 6, iter = 15000, warmup = 5000, control = list(adapt_delta = 0.95)).

Univariate maximal FID model: brm (FID ~ VODI + Engaged + Habitat + Height + Number of neighbors + nearest neighbor flee first + external events within 5 min + Observer*Trial number + (1|Date) + (1+ VODI + Observer*Trial number|p|ID), data = FID, family = log-normal, prior = prior, chains = 6, iter = 15,000, warmup = 5000, control = list(adapt_delta = 0.95)).

Bivariate model:

Mod.VOD ≤ bf (VOD ~ VODD + Compatibility + Habitat + Height + Number of neighbors + Nearest neighbor flee first + External events within 5 min + Observer*Trial number + (1|Date) + (1+ VODD + Observer*Trial number|p|ID) + log-normal().

Mod.FID ≤ bf (FID ~ VODI + Engaged + Habitat + Height + Number of neighbors + nearest neighbor flee first + external events within 5 min + Observer*Trial number + (1|Date) + (1+ VODI + Observer*Trial number|p|ID) + log-normal().

Bivariate.Mod ≤ brm (mod.vod + mod.fid, data = fid, prior = prior, chains = 6, iter = 15,000, warmup = 5000, control = list(adapt_delta = 0.95)).

## Supplementary Material

aaz0870_SM.pdf

## SUPPLEMENTARY MATERIALS

Supplementary material for this article is available at http://advances.sciencemag.org/cgi/content/full/6/28/eaaz0870/DC1
