# Supplementary material for: Habituation is not neutral or equal: Individual differences in tolerance suggest an overlooked personality trait
Source: Sci Adv. 2020 Jul 8;6(28):eaaz0870. doi: 10.1126/sciadv.aaz0870 (PMC7343399; doi:10.1126/sciadv.aaz0870)
Supplement: aaz0870_SM.pdf [file aaz0870_SM.pdf]

Science Advances
AAAS

advances.sciencemag.org/cgi/content/full/6/28/eaaz0870/DC1

Supplementary Materials for

Habituation is not neutral or equal: Individual differences in tolerance suggest an overlooked personality trait

Andrew T. L. Allan*, Annie L. Bailey, Russell A. Hill

*Corresponding author. Email: a.t.l.allan@durham.ac.uk

Published 8 July 2020, *Sci. Adv.* **6**, eaaz0870 (2020)
DOI: 10.1126/sciadv.aaz0870

This PDF file includes:

Tables S1 to S3
Texts S1 and S2
References

Table S1. Contextual variables that could be major drivers of VOD and FID responses in habituated chacma baboons with examples from literature.

Factors	Link to sensory capacity/FID/personality	Example
**Response variable: VOD**		
Observer (pseudo-predator) identity, X_1	Unfamiliar observer considered a greater threat, leading to increased risk-perception and tendency to visually orient, resulting in longer VOD	(*9*, *12*)
Trial number, X_2	(i) Increase or decrease in VOD with trial number indicative of habituation or sensitization (respectively) to FID approach methodology<br>(ii) Consistent individual VOD response through time indicates personality trait	(i) (*49*, *60*)<br>(ii) (*22*, *23*)
Compatibility: Not engaged (Looking/Not looking), Engaged (Not Looking) X_3	Looking may enable animals to collect multiple types of information concurrently; in addition, being “not engaged” may afford focal animals a greater sensory capacity for detection. As a result, individuals looking as approach commences will visually orient toward approaching observer sooner resulting in longer VOD; engaged should yield shorter VOD.	(*41*, *61*)
Habitat (open/closed), X_4	(i) “Open” habitats may afford individuals greater visibility increasing likelihood of attending to approaching observer quicker, resulting in longer VOD<br>(ii) Open habitats are generally considered safer for baboons as they permit earlier detection and avoidance of predators, therefore risk perception could be lower, reducing tendency to visual orient towards approaching observer, resulting in shorter VOD<br>(iii) Open habitats may increase risk perception as focal animals are less concealed from potential threats, increasing tendency to visually orient towards approaching observer, resulting in longer VOD.<br>(iv) “Open” habitats have lower refuge availability which may increase risk perception, resulting in longer VOD	(i) (*41*)<br>(ii) (*62*)<br>(iii) (*63*)<br>(iv) (*62*–*64*)
Height (ground/above ground), X_5	“Above ground” may afford individuals greater visibility, resulting in longer VOD. In this context “above ground” is <50cm from ground level and is unlikely to qualify as potential refuge (see (*62*)) and therefore should not influence risk-perception.	(*41*)
Number of neighbors within 5m, X_6	(i) As number of neighbors increase, the likelihood of a neighbor visually orienting towards the approacher increases, i.e., collective detection, which could result in longer VOD<br>(ii) As number of neighbors increase, the likelihood of predation decreases reducing risk perception and the tendency to visually orient towards the approach observer, resulting in shorter VOD<br>(iii) Increasing number of neighbors may mask both the visual and audible cues associated with the observer’s approach, resulting shorter VOD, e.g., neighbors draw visual attention away from observer, or noises from neighbours mask the sounds of observer’s footsteps during approach.	(i) Collective detection: (*65*)<br>(ii) Dilution effect: (*66*, *67*)<br>(iii) (*68*)
Neighbor flight, X_7	Local conspecifics initiating flight before the focal animal will increase risk perception and evoke vigilance. Both factors could lead to focal animals visually orienting toward approaching observer sooner, resulting in longer VOD.	Neighbor flight may provide conspecifics information on local threats (*69*)
External factors (local alarms, aggressions within 5 min), X_8	(i) Localized threatening stimuli leads to increased risk perception and tendency to visually orient, resulting in longer VOD<br>(ii) Localized visual and audible stimuli may reallocate some of the focal animal’s finite attention, resulting in longer VOD	(i) (*19*)<br>(ii) (*68*)
**Response variable: FID**		
VODI, X_9	When visual orientation interval (distance between VOD and FID) is long, focal animals will flee sooner, resulting in longer FID.	F.E.A.R hypothesis: (*26*)
Engaged/Not engaged, X_{10}	FID will be higher if focal animal was engaged at the start of the approach, as flight costs are higher because of interrupted social time (i.e., grooming) or loss of food patch (i.e., foraging)	FID lower during agonistic, reproductive or social interactions (*70*, *71*). Animals in higher quality food patches are associated with shorter flight distances (*72*–*74*).
Observer (pseudo-predator) identity, X_1	Unfamiliar observer is considered a greater threat; therefore, FID should be greater for unfamiliar observer	(*9*, *12*)
Trial number, X_2	(i) Increase or decrease in FID with trial number indicative of sensitization or habituation (respectively) to FID approach methodology<br>(ii) Consistent FID response through time indicates personality trait	(i) (*49*, *60*)<br>(ii) (*22*, *23*)

Habitat (open/closed), X_4	(i) Open habitats are generally considered safer for baboons as they permit earlier detection and avoidance of predators; therefore, risk perception could be lower, resulting in shorter FID<br>(ii) Open habitats may increase risk perception as focal animals are less concealed from potential threats, resulting in longer FID<br>(iii) Open habitats have lower refuge availability which may increase risk perception, resulting in longer FID	(i) (*62*)<br>(ii) (*63*, *64*)<br>(iii) (*62–64*)
Number of neighbors within 5m, X_6	(i) Risk diluted with greater number of neighbors; therefore, FID should decrease as number of neighbors increases.<br>(ii) Increasing number of neighbors increases localised visual and audible stimuli, and therefore may reallocate some of the focal animal's finite attention resulting in decreased FID	(i) Group size effect: (*75*)<br>(ii) (*68*)
Neighbor flight, X_7	Local conspecifics initiating flight before the focal animal will increase risk perception; and therefore, increase FID.	Focal animals may flee sooner based on neighbor flight (*69*)
External factors (local alarms, aggressions within 5 min), X_8	(i) Localized threatening stimuli leads to increased risk perception and therefore increased FID<br>(ii) Localised visual and audible stimuli may reallocate some of the focal animal's finite attention therefore decreasing FID	(i) (*19*)<br>(ii) (*68*)

Table S2. Summary of intra-class correlation and convergent validity validation analyses for independent VOD and FID data points. For both univariate and bivariate models, VOD data was taken from even numbered trials (e.g., trial number 2, 4, 6 etc) and FID data was taken from odd numbered trials, creating independent datasets. Calculations for ICC and convergent validity analyses followed the same protocol as outlined in *Statistical analyses* for the main dataset. Lower and upper HDI refers to highest density intervals for posterior samples at 95% intervals.

Model type	Mean	Lower HDI	Upper HDI
**Univariate**			
VOD ICC	0.378	0.207	0.546
FID ICC	0.613	0.507	0.717
**Bivariate**			
VOD ICC	0.345	0.201	0.495
FID ICC	0.591	0.491	0.689
Convergent validity	0.773	0.577	0.94

Table S3. Summary start distances for each individual

ID	Age-sex class	Number of approaches	Min start distance	Max start distance	Average start distance
ARL	J2M	24	2.532	9.587	5.103
ATH	AF	24	3.076	13.161	6.038
BAM	J1M	24	2.941	9.573	4.749
BIX	J2F	24	3.112	9.494	5.471
BLO	AM	24	3.483	9.261	5.351
BOU	AF	24	2.837	10.323	5.105
BOX	AF	24	2.607	9.127	4.973
BRA	AF	24	3.114	10.677	5.271
BRU	AF	24	3.115	9.868	5.220
BUR	J2M	24	3.305	9.687	5.652
CAR	ADF	24	3.289	9.843	5.312
CLO	J1F	24	2.919	8.42	5.165
COR	ADF	24	3.232	9.333	5.639
CRO	ADM	24	3.31	7.571	5.088
DAN	J1F	24	3.087	11.842	5.668
DAV	AM	24	3.412	9.922	5.250
DIC	J1M	24	2.779	9.932	5.411
DIL	J2M	24	2.91	8.466	5.891
DIN	J1M	24	2.817	10.35	5.503
ECH	ADF	24	2.907	8.948	4.696
EGO	AM	24	3.329	11.272	6.162
ELA	AF	24	3.433	13.646	7.909
EVI	AF	24	3.001	10.586	5.883
FLE	AM	24	3.609	10.26	5.675
FUN	J1F	24	2.889	9.993	5.141
GRO	J1M	24	3.026	10.889	5.192
GRU	AF	24	2.937	10.995	5.476
HEA	AF	24	3.14	12.728	6.694
HEN	ADM	24	2.739	10.771	5.442
HUN	J2M	24	3.001	8.138	4.970
JAC	J2M	24	3.075	14.242	5.819
JOS	AM	24	3.37	10.806	6.316
LAR	J1M	24	3.17	10.861	5.632
LAT	J1M	24	3.342	9.447	5.082
LOB	AF	24	2.748	8.693	5.001
LUK	J3M	24	2.73	9.272	5.223
MAN	AF	24	3.504	13.452	6.637
MEL	AF	24	3.239	12.28	7.024
MOU	J1M	24	3.007	8.644	4.869
MUR	ADF	24	3.392	10.478	5.444
NAT	ADM	24	3.507	11.524	5.668
NIC	J1M	24	3.407	11.479	5.933
NOR	AF	24	4.132	12.279	7.237
NOS	AM	24	3.17	17.082	6.589
PIX	AF	24	3.265	9.964	5.853
PON	J1M	24	2.747	9.63	4.897
PRA	J3M	24	2.69	8.85	5.180
PRE	ADM	24	2.98	10.436	5.432
RHO	ADF	24	2.762	8.535	4.909
RIP	AF	24	3.359	10.115	6.453
SAC	J2F	24	2.922	8.404	5.194
SAN	J2F	24	2.751	10.055	5.357
SCA	AF	24	2.946	9.306	5.075
SCO	J1M	24	2.769	9.309	5.040
SEX	AM	24	3.018	11.105	6.885
SIL	AF	24	3.174	33.864	7.243
SLI	AF	24	3.192	10.142	5.570
STE	J1M	24	3.008	8.884	5.465
STI	J1F	24	3.171	9.274	4.950
STR	J2M	24	2.985	9.164	5.596
TER	AF	24	4.262	17.583	8.864
THI	AF	24	3.035	8.541	5.794
TIL	J2F	24	2.989	9.17	5.371
TON	J1F	24	3.407	9.413	5.534
TRI	AF	24	2.581	7.843	4.858
TRU	J1F	24	2.88	11.377	5.179
TUP	AF	24	3.051	8.863	5.724
YOD	J1F	24	3.048	11.576	5.887
YOL	AF	24	4.615	22.162	11.053

Text S1. Age-sex class categories and descriptions
Female baboons:
AF (Adult female) – Attainment of full body size, either cycling regularly, pregnant or lactating. Nipples also enlarge and elongated from suckling infants.
ADF (Adolescent Female) – Nearly adult female size, with the onset of the first sexual swellings. If visible, nipples are much smaller and button-like than that of an adult female.

Male baboons:
AM (Adult male) – All secondary sexual characteristics fully grown, musculature (most noticeably in chest and rump) expands to full adult size.
ADM (Adolescent Male) – Massive growth in secondary sexual characteristics; testes expand, canines and mane grow longer, body size increases to near that of an adult male.
J3M (Juvenile 3 Males Only) – Body size that of an adult female, muzzle further extended to nearly that of an adult male. Testes start to expand and are clearly visible. Mane becomes noticeable.

Juvenile baboons of both sexes:
J2M/F (Juvenile 2) – Little demarcation from previous period, with greater body size. Hair becomes darker, changing to a more adult grey/brown colouration.
J1M/F (Juvenile 1) – Little demarcation from infants, but fully weaned and nutritionally independent. Muzzle starts becoming more elongated and pronounced. Pelage is still lighter than in juvenile 2. Male/female distinction based on genitalia and noticeable absence/presence of a separation in the callosities.

Text S2. Descriptions of the various habitat types categorised in this study
Habitat type – the Lajuma field site and surrounding properties contain a range of habitat types that can be broadly classified into several categories: forest, woodland, bush, camp, farm, marsh, grassland, rock and cliff. These habitat types will vary in their respective structure and therefore influence visibility to a different extent.

"Closed" habitats
Forest: An area composed largely of trees with overlapping crowns forming 60-100% cover. Trees will be mostly tall providing extensive and near continuous shade.
Woodland: Canopy is more open than forest, with 25-60% cover, allowing sunlight to penetrate between the trees. Woodlands may support an understory of shrubs, herbs, or grasses.
Bush: An area where shrubs or are the dominant vegetation. A shrub is a woody perennial plant, smaller than a tree, with several major branches arising near the base of the stem. Areas of extensive tree regeneration, i.e., saplings, can also qualify as bush.

"Open" habitats
Grassland or savannah: Open area covered predominantly with grass. These areas may be devoid of trees entirely but can also contain widely spaced trees with a minimum of 5% cover to a maximum of 25% cover.
Marshland: Exclusively found in flat regions along permanent water streams on peat. Vegetation components of marshlands include reeds, sedges, and grasses.
Rocks: Areas where ground predominantly consists of rocks and boulders, rather than soil.
Road: Dirt roads that run through the study area. Very little traffic (less than 5 vehicles a day) and always at low speeds (less than 10mph). Road use often offers a localised enhancement in visibility for the baboons.
Camp: Used or disused human settlements on Lajuma and neighbouring properties.
Farm: Ottosdal Macadamia farm or area around Ottosdal farmhouse.

REFERENCES AND NOTES

1. D. T. Blumstein, Habituation and sensitization: New thoughts about old ideas. *Anim. Behav.* **120**, 255–262 (2016).

2. P. Bertolani, C. Boesch, Habituation of wild chimpanzees (*Pan troglodytes*) of the South Group at Taï Forest, Côte d'Ivoire: Empirical measure of progress. *Folia Primatol.* **79**, 162–171 (2008).

3. L. M. Fedigan, Ethical issues faced by field primatologists: Asking the relevant questions. *Am. J. Primatol.* **72**, 754–771 (2010).

4. A. le Roux, M. I. Cherry, M. B. Manser, The audience effect in a facultatively social mammal, the yellow mongoose, *Cynictis penicillata*. *Anim. Behav.* **75**, 943–949 (2008).

5. R. J. Welch, A. le Roux, M. B. Petelle, S. Périquet, The influence of environmental and social factors on high- and low-cost vigilance in bat-eared foxes. *Behav. Ecol. Sociobiol.* **72**, 29 (2018).

6. B. M. Titus, M. Daly, D. A. Exton, Do reef fish habituate to diver presence? Evidence from two reef sites with contrasting historical levels of SCUBA intensity in the bay islands, honduras. *PLOS ONE* **10**, e0119645 (2015).

7. B. B. Smuts, *Sex and Friendship in Baboons* (Harvard University Press, 1999).

8. K. T. Hanson, E. P. Riley, Beyond neutrality: The human–primate interface during the habituation process. *Int. J. Primatol.* **39**, 852–877 (2017).

9. L. Alcayna-stevens, Habituating field scientists. *Soc. Stud. Sci.* **46**, 833–853 (2016).

10. M. Candea, Habituating meerkats and redescribing animal behaviour science. *Theory Cult. Soc.* **30**, 105–128 (2013).

11. S. D. Gosling, From mice to men: What can we learn about personality from animal research? *Psychol. Bull.* **127**, 45–86 (2001).

12. E. A. Williamson, A. T. C. Feistner, Habituating primates: Process, technique, variables and ethics, in *Field and Laboratory Methods in Primatology: A Practical Guide*, J. M. Setchell, D. J. Curtis, Eds. (Cambridge Univ. Press, ed. 2, 2011), pp. 33–50.

13. K. M. Jack, B. B. Lenz, E. Healan, S. Rudman, V. A. M. Schoof, L. Fedigan, The effects of observer presence on the behavior of *Cebus capucinus* in Costa Rica. *Am. J. Primatol.* **70**, 490–494 (2008).

14. P. McDougall, Is passive observation of habituated animals truly passive? *J. Ethol.* **30**, 219–223 (2012).

15. S. K. Iredale, C. H. Nevill, C. K. Lutz, The influence of observer presence on baboon (*Papio spp*.) and rhesus macaque (*Macaca mulatta*) behavior. *Appl. Anim. Behav. Sci.* **122**, 53–57 (2010).

16. M. C. Crofoot, T. D. Lambert, R. Kays, M. C. Wikelski, Does watching a monkey change its behaviour? Quantifying observer effects in habituated wild primates using automated radiotelemetry. *Anim. Behav.* **80**, 475–480 (2010).

17. A. J. Carter, H. H. Marshall, R. Heinsohn, G. Cowlishaw, How not to measure boldness: Novel object and antipredator responses are not the same in wild baboons. *Anim. Behav.* **84**, 603–609 (2012).

18. D. Réale, S. M. Reader, D. Sol, P. T. McDougall, N. J. Dingemanse, Integrating animal temperament within ecology and evolution. *Biol. Rev. Camb. Philos. Soc.* **82**, 291–318 (2007).

19. D. T. Blumstein, D. S. M. Samia, T. Stankowich, W. E. Cooper, Escaping from predators, *An Integrative View of Escape Decisions*, W. E. Cooper, D. T. Blumstein, Eds. (Cambridge Univ. Press, 2015), pp. 405–419.

20. W. E. Cooper Jr., W. G. Frederick, Optimal flight initiation distance. *J. Theor. Biol.* **244**, 59–67 (2007).

21. R. C. Ydenberg, L. M. Dill, The economics of fleeing from predators. *Adv. Study Behav.* **16**, 229–249 (1986).

22. A. J. Carter, R. Heinsohn, A. W. Goldizen, P. A. Biro, Boldness, trappability and sampling bias in wild lizards. *Anim. Behav.* **83**, 1051–1058 (2012).

23. M. B. Petelle, D. E. McCoy, V. Alejandro, J. G. A. Martin, D. T. Blumstein, Development of boldness and docility in yellow-bellied marmots. *Anim. Behav.* **86**, 1147–1154 (2013).

24. A. J. Carter, W. E. Feeney, H. H. Marshall, G. Cowlishaw, R. Heinsohn, Animal personality: What are behavioural ecologists measuring? *Biol. Rev. Camb. Philos. Soc.* **88**, 465–475 (2013).

25. T. M. Houslay, A. J. Wilson, Avoiding the misuse of BLUP in behavioural ecology. *Behav. Ecol.* **28**, 948–952 (2017).

26. D. T. Blumstein, Flush early and avoid the rush: A general rule of antipredator behavior? *Behav. Ecol.* **21**, 440–442 (2010).

27. Y. Yao, A. Vehtari, D. Simpson, A. Gelman, Using stacking to average bayesian predictive distributions (with Discussion). *Bayesian Anal.* **13**, 917–1007 (2018).

28. M. A. Stoffel, S. Nakagawa, H. Schielzeth, rptR: Repeatability estimation and variance decomposition by generalized linear mixed-effects models. *Methods Ecol. Evol.* **8**, 1639–1644 (2017).

29. T. K. Koo, M. Y. Li, A guideline of selecting and reporting intraclass correlation coefficients for reliability research. *J. Chiropr. Med.* **15**, 155–163 (2016).

30. J. S. Strong, R. B. Weladji, Ø. Holand, K. H. Røed, M. Nieminen, Personality and fitness consequences of flight initiation distance and mating behavior in subdominant male reindeer (*Rangifer tarandus*). *Ethology* **123**, 484–492 (2017).

31. A. K. Boon, D. Réale, S. Boutin, The interaction between personality, offspring fitness and food abundance in North American red squirrels. *Ecol. Lett.* **10**, 1094–1104 (2007).

32. R. Kliegl, P. Wei, M. Dambacher, M. Yan, X. Zhou, Experimental effects and individual differences in linear mixed models: Estimating the relationship between spatial, object, and attraction effects in visual attention. *Front. Psychol.* **1**, 238 (2011).

33. M. J. Merrick, J. L. Koprowski, Should we consider individual behavior differences in applied wildlife conservation studies? *Biol. Conserv.* **209**, 34–44 (2017).

34. M. Díaz, A. P. Møller, E. Flensted-Jensen, T. Grim, J. D. Ibáñez-Álamo, J. Jokimäki, G. Markó, P. Tryjanowski, The geography of fear: A latitudinal gradient in anti-predator escape distances of birds across Europe. *PLOS ONE* **8**, e64634 (2013).

35. A. Frid, L. M. Dill, Human-caused disturbance stimuli as a form of predation risk. *Ecol. Soc.* **6**, 11 (2002).

36. K. Uchida, K. K. Suzuki, T. Shimamoto, H. Yanagawa, I. Koizumi, Decreased vigilance or habituation to humans? Mechanisms on increased boldness in urban animals. *Behav. Ecol.* **30**, 1583–1590 (2019).

37. D. S. M. Samia, D. T. Blumstein, Phi index: A new metric to test the flush early and avoid the rush hypothesis. *PLOS ONE* **9**, e113134 (2014).

38. F.-R. Favreau, A. W. Goldizen, H. Fritz, S. P. Blomberg, E. C. Best, O. Pays, Within-population differences in personality and plasticity in the trade-off between vigilance and foraging in kangaroos. *Anim. Behav.* **92**, 175–184 (2014).

39. F. Barnier, P. Duncan, H. Fritz, P. Blanchard, D. I. Rubenstein, O. Pays, Between-gender differences in vigilance do not necessarily lead to differences in foraging-vigilance tradeoffs. *Oecologia* **181**, 757–768 (2016).

40. M. Watson, N. J. Aebischer, W. Cresswell, Vigilance and fitness in grey partridges *Perdix perdix*: The effects of group size and foraging-vigilance trade-offs on predation mortality. *J. Anim. Ecol.* **76**, 211–221 (2007).

41. A. T. L. Allan, R. A. Hill, What have we been looking at? A call for consistency in studies of primate vigilance. *Am. J. Phys. Anthropol.* **165**, 4–22 (2018).

42. T. H. Clutton-Brock, A. Maccoll, P. Chadwick, D. Gaynor, R. Kansky, J. D. Skinner, Reproduction and survival of suricates (*Suricata suricatta*) in the southern Kalahari. *Afr. J. Ecol.* **37**, 69–80 (2001).

43. N. R. Jordan, F. Mwanguhya, S. Kyabulima, P. Rüedi, M. A. Cant, Scent marking within and between groups of wild banded mongooses. *J. Zool.* **280**, 72–83 (2010).

44. L. Bejder, A. Samuels, H. Whitehead, H. Finn, S. Allen, Impact assessment research: Use and misuse of habituation, sensitisation and tolerance in describing wildlife responses to anthropogenic stimuli. *Mar. Ecol. Prog. Ser.* **395**, 177–185 (2009).

45. G. Roberts, Why individual vigilance declines as group size increases. *Anim. Behav.* **51**, 1077–1086 (1996).

46. K. Nowak, A. Le Roux, S. A. Richards, C. P. J. Scheijen, R. A. Hill, Human observers impact habituated samango monkeys' perceived landscape of fear. *Behav. Ecol.* **25**, 1199–1204 (2014).

47. A. L. de Raad, R. A. Hill, Topological spatial representation in wild chacma baboons (*Papio ursinus*). *Anim. Cogn.* **22**, 397–412 (2019).

48. F. Dumont, C. Pasquaretta, D. Réale, G. Bogliani, A. von Hardenberg, Flight initiation distance and starting distance: Biological effect or mathematical artefact? *Ethology* **118**, 1051–1062 (2012).

49. A. M. Runyan, D. T. Blumstein, Do individual differences influence FLIGHT initiation distance? *J. Wildl. Manage.* **68**, 1124–1129 (2004).

50. P.-C. Bürkner, brms : An R package for Bayesian multilevel models using stan. *J. Stat. Softw.* **80**, 1–28 (2017).

51. R Core Team, R: A language and environment for statistical computing, version 3.6.1. *R Found. Stat. Comput* (Vienna, Austria, 2019).

52. Stan Development Team, RStan: The R interface to Stan, *R Found. Stat. Comput.* (Vienna, Austria, 2019).

53. R Core Team, Rtools software, Version 35, *R Found. Stat. Comput.* (Vienna, Austria, 2018).

54. A. Gelman, D. B. Rubin, Inference from iterative simulation using multiple sequences. *Stat. Sci.* **7**, 457–472 (1992).

55. D. S. M. Samia, F. Nomura, D. T. Blumstein, Do animals generally flush early and avoid the rush? A meta-analysis. *Biol. Lett.* **9**, 20130016 (2013).

56. S. Chamaillé-Jammes, D. T. Blumstein, A case for quantile regression in behavioral ecology: Getting more out of flight initiation distance data. *Behav. Ecol. Sociobiol.* **66**, 985–992 (2012).

57. N. C. Bonnot, A. J. M. Hewison, N. Morellet, J.-M. Gaillard, L. Debeffe, O. Couriot, B. Cargnelutti, Y. Chaval, B. Lourtet, P. Kjellander, C. Vanpé, Stick or twist: Roe deer adjust their flight behaviour to the perceived trade-off between risk and reward. *Anim. Behav.* **124**, 35–46 (2017).

58. A. Vehtari, A. Gelman, J. Gabry, Y. Yao, loo: Efficient leave-one-out cross-validation and WAIC for Bayesian models, R Package. version 2.0.0; (2018).

59. A. Vehtari, D. P. Simpson, Y. Yao, A. Gelman, Limitations of "Limitations of bayesian leave-one-out cross-validation for model selection". *Comput. Brain Behav.* **2**, 22–27 (2019).

60. M. Carrete, J. L. Tella, Individual consistency in flight initiation distances in burrowing owls: A new hypothesis on disturbance-induced habitat selection. *Biol. Lett.* **6**, 167–170 (2010).

61. J. Krause, J.-G. J. Godin, Influence of prey foraging posture on flight behavior and predation risk: Predators take advantage of unwary prey. *Behav. Ecol.* **7**, 264–271 (1996).

62. G. Cowlishaw, Trade-offs between foraging and predation risk determine habitat use in a desert baboon population. *Anim. Behav.* **53**, 667–686 (1997).

63. M. J. Camp, J. L. Rachlow, B. A. Woods, T. R. Johnson, L. A. Shipley, When to run and when to hide: The influence of concealment, visibility, and proximity to refugia on perceptions of risk. *Ethology* **118**, 1010–1017 (2012).

64. T. Stankowich, Ungulate flight responses to human disturbance: A review and meta-analysis. *Biol. Conserv.* **141**, 2159–2173 (2008).

65. S. L. Lima, Back to the basics of anti-predatory vigilance: the group-size effect. *Anim. Behav.* **49**, 11–20 (1995).

66. W. D. Hamilton, Geometry for the selfish herd. *J. Theor. Biol.* **31**, 295–311 (1971).

67. I. Vine, Risk of visual detection and pursuit by a predator and the selective advantage of flocking behaviour. *J. Theor. Biol.* **30**, 405–422 (1971).

68. A. A. Y. Chan, P. Giraldo-perez, S. Smith, D. T. Blumstein, Anthropogenic noise affects risk assessment and attention: the distracted prey hypothesis. *Biol. Lett.* **6**, 458–461 (2010).

69. S. L. Lima, Collective detection of predatory attack by birds in the absence of alarm signals. *J. Avian Biol.* **25**, 319–326 (1994).

70. O. Brick, Fighting behaviour, vigilance and predation risk in the cichlid fish Nannacara anomala. *Anim. Behav.* **56**, 309–317 (1998).

71. W. E. Cooper, Variation in escape behavior among individuals of the striped plateau lizard *Sceloporus virgatus* may reflect differences in boldness. *J. Herpetol.* **43**, 495–502

(2009).

72. W. E. Cooper, V. Pérez-Mellado, D. Hawlena, Magnitude of food reward affects escape behavior and acceptable risk in Balearic lizards, *Podarcis lilfordi*. *Behav. Ecol.* **17**, 554–559

(2006).

73. P. A. Lagos, A. Meier, L. O. Tolhuysen, R. A. Castro, F. Bozinovic, L. A. Ebensperger, Flight initiation distance is differentially sensitive to the costs of staying and leaving food patches in a small-mammal prey. *Can. J. Zool.* **87**, 1016–1023 (2009S).

74. G. J. Scrimgeour, J. M. Culp, Nordic society Oikos foraging and evading predators: The effect of predator species on a behavioural Trade-off by a Lotic Mayfly. *Oikos.* **69**, 71–79 (1994).

75. T. K. Matson, A. W. Goldizen, D. A. Putland, Factors affecting the vigilance and flight behaviour of impalas. *South African J. Wildl. Res.* **35**, 1–10 (2005).
